# Understanding Multi-Level Factors Impacting Digital Health Literacy in the Deep South of the United States

**DOI:** 10.3390/ijerph22010041

**Published:** 2024-12-31

**Authors:** Tanvi V. Padalkar, Keyonsis Hildreth, Gabrielle B. Rocque, Stacey A. Ingram, Omari Whitlow, Dan Chu, Connie C. Shao, Courtney P. Williams, Claudia M. Hardy, Chao-Hui Sylvia Huang, Nicole L. Henderson

**Affiliations:** 1 Department of Medicine, Division of Hematology and Oncology, University of Alabama at Birmingham, Birmingham, AL 35233, USA; tpadalkar@uabmc.edu (T.V.P.); kjhildreth@uabmc.edu (K.H.); grocque@uabmc.edu (G.B.R.); saadewakun@uabmc.edu (S.A.I.); omariwhitlow@uabmc.edu (O.W.); chardy@uab.edu (C.M.H.); 2 O’Neal Comprehensive Cancer Center, Birmingham, AL 35233, USA; courtneywilliams@uabmc.edu; 3 Department of Surgery, University of Alabama at Birmingham, Birmingham, AL 35233, USA; dchu@uabmc.edu (D.C.); cshao@uabmc.edu (C.C.S.); 4 Department of Medicine, Division of General Internal Medicine and Population Science, University of Alabama at Birmingham, Birmingham, AL 35233, USA; 5 Department of Medicine, Division of Gerontology, Geriatrics and Palliative Care, University of Alabama at Birmingham, Birmingham, AL 35233, USA; chhuang@uabmc.edu

**Keywords:** digital health literacy, community health advisors, Taplin Multi-Level Model

## Abstract

As healthcare and health services become increasingly digitized, individuals with low digital health literacy (DHL) may experience inequitable care and outcomes. We explored factors impacting DHL and recommendations for improvement from community health coordinators and advisors (CHAs) in Alabama and Mississippi in United States. Semi-structured interviews were conducted with CHAs to gather insights on their perspectives on and experiences with DHL. Interviews were transcribed and analyzed using a grounded coding schema, with key barriers and recommendations mapped onto the Taplin Multi-Level Intervention model to help identify influences across various levels. Thirty-two CHAs participated, predominantly female (94%) and Black or African American (94%). At the individual level, factors involved demographic characteristics, increased patient workload, and attitudes towards technology. Social support was captured at the relationships level. At the provider level, provider–patient communication and injustices were involved, compounded by health system infrastructure and culture at the practice-setting level. Resource landscape and shared knowledge and beliefs were significant at the community level. The COVID-19 pandemic further highlighted these challenges at the societal level. CHAs provided targeted recommendations for addressing barriers at each level. CHAs identified multi-level factors contributing to DHL and emphasized levels based on comprehensive interventions.

## 1. Introduction

The integration of digital technologies into cancer care delivery has great potential for improving population health, along with individual health and well-being [1,2]. In the last decade, previous studies have demonstrated the positive health and psychosocial outcomes of technologies such as the electronic health record, patient portals, electronic Patient Reported Outcomes (ePROs), wearable devices, telehealth, and telemedicine [1,3,4,5,6,7,8,9,10]. Digital technologies also have been shown to provide informational support to both patients with cancer and their caregivers [11]. These favorable outcomes have led to the inclusion of ePROs as a requirement for participation in Centers for Medicaid and Medicare Services (CMS) Enhancing Oncology Model (EOM), a value-based healthcare payment reform demonstration project [12]. Thus, a continued increase in implementation and utilization of these technologies is likely.

In this context, careful attention must be paid to differences in uptake for diverse patient groups, as this could negatively impact historically disadvantaged populations and contribute further to existing health inequities [13,14,15]. Adoption and regular utilization of healthcare technology is low for patients with cancer who are older, of low socioeconomic status, non-English speaking, and individuals with low health literacy and lack of access to technological resources [11,14,16,17,18,19]. Sociodemographic factors tied to age, education, income, along with perceived health and social isolation also predicted internet access, with digital skills broadly influencing internet use for healthcare needs [20,21]. DHL was expressed to positively influence medical decision making, mental and psychological states, and quality of life [3]. To describe these gaps, the concept of digital health literacy (DHL) has recently emerged, which recognizes variations in skill required for using technological resources and refers to the individual’s ability to seek, process, and appraise health information through electronic sources and in digital contexts and apply the knowledge and skills to addressing health problems [22]. The use of digital technologies in cancer care—where patients’ symptom burden is often high—offers avenues for empowering patients in symptom management or fulfilling their informational needs [8,9,23]. Given the pertinent impacts of DHL and implementation of digital interventions, it is crucial to understand the community-based landscape of digital health in cancer care. This is particularly impactful given the gap in literature regarding populations in rural locations within the United States (US) such as in the Deep South, which is characterized by states in the south-eastern US (i.e., Alabama, Florida, Georgia, Louisiana, Mississippi, North Carolina, South Carolina, Tennessee, and Texas) with majority Black counties [24]. In rural US, the Federal Communication Commission (FCC) estimates express that around 9.3 million rural residents have inadequate broadband service [25]. As reported within Alabama, broadband subscription in Alabama was 79.9% and 80.3% in Mississippi, along with 65.8% in the Black Belt region of the Deep South, US, which is lower than the national rate of 85.2% [26].

The foundation of DHL is built upon health literacy, digital literacy, and basic literacy skills [27]. Therefore, it is essential to acknowledge the underlying issues and appreciate the broader socioeconomic and cultural factors at play that contribute to each of these discrepancies in DHL. In Alabama and Mississippi, community health coordinators and advisors (CHAs) are trusted individuals in their communities who are trained to serve as a bridge between the community and clinical setting, with their work supporting health education, treatment adherence, health system navigation, and linkage to social services for individuals receiving cancer care [28,29]. Thus, CHAs are ideally situated to link individuals to the healthcare system and can provide some insights from the healthcare arena and broader community. CHAs can contextualize on-the-ground experiences with digital health literacy and promote the equitable implementation of technological health care services that seek to address gaps in cancer care access, feasibility, and sustainability in the Deep South and nationwide [29,30]. In this qualitative study, we explored the barriers and their specific recommendations to address those barriers, which impact DHL among patients in the Deep South, as perceived by CHAs.

## 2. Materials and Methods

### 2.1. Study Design, Participants, and Setting

This was part of a broader cross-sectional qualitative study that used grounded theory to explore CHA experiences with and perspectives on DHL among patients, alongside multi-level regional barriers to health technology, and potential solutions to address barriers based on the Taplin multi-level factors [31]. The sampling frame consisted of CHAs from the University of Alabama at Birmingham (UAB) and the Mitchell Cancer Institute at the University of South Alabama (USA) that serve patient populations throughout Alabama and Mississippi. All the community health advisors attending the 2023 Community Dissemination Institute, held by the O’Neal Comprehensive Cancer Center, were identified using convenience and snowball sampling and approached by a research team member with an invitation to participate in a 30–60 min semi-structured interview from March to November 2023. Interviews were conducted in person, over telephone, or via Zoom by a clinical research coordinator with a background in public health and health education (KH). Interviews were audio recorded and transcribed using an independent transcription service (https://www.rev.com (accessed starting on 6 March 2023)). The UAB Institutional Review Board approved this study (IRB#300010079).

### 2.2. Data Analysis

The semi-structured interview guide was developed to reflect on the Taplin Multi-Level Intervention model and approved by a multidisciplinary research team including a medical oncologist (GR) and anthropologist (NH) [31]. Topical foci on DHL of the interviews included (1) digitalization of healthcare, (2) barriers to technology as observed by CHAs, (3) digital health literacy, and (3) perceptions of community-level support to address technological challenges. Interview transcripts were then analyzed using a grounded theoretical approach to understand DHL barriers and facilitators at each level of the multi-level factors of the Taplin model in relation to digital health literacy [31]. A medical anthropologist (NH) led the initial inductive open coding and categorization of codes. This coding scheme was reviewed and formalized by the research team (KH, GR, NH) and was followed by a second round of focused coding by three independent coders (NH, TP, OW). The research team continuously discussed any coding discrepancies to ensure accurate representation of CHA perspectives. This continued until a satisfactory interrater reliability level was demonstrated (Κ > 0.7).

**Figure 1:**
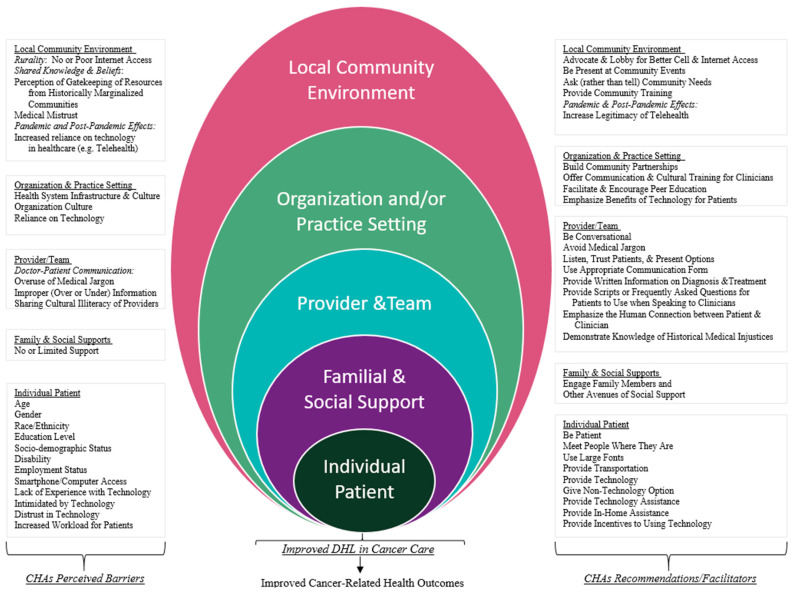
CHAs perceived facilitators/recommendations to address barriers to digital health literacy according to the Taplin Multi-Level Intervention model.

Within the analysis, the coding scheme was mapped onto the Taplin Multi-Level Intervention model to tease out the complexity of DHL barriers and facilitators at the appropriate level of impact. The Taplin model identifies targets of intervention along the cancer care continuum that are influential in healthcare delivery and presents them as ecological levels with various unique mechanism factors considered with the levels [31]. The levels focused on in the Taplin model and applied to the coding scheme included the following: individual patient, family and social support, provider/team, organization and/or practice setting, local community environment, state health policy environment, and national health policy environment [31]. The research team and coders (GR, KH, NH, TP, OW) reviewed and mapped the coding scheme to the Taplin model levels—based on Taplin’s definitions of the levels—and focused on barriers and recommendations associated with DHL at each ecological level. The team iteratively reviewed the Taplin Multi-Level Intervention model and coding schema mapping to ensure proper representation of DHL experiences as influenced by the levels and described by the CHAs. For the purposes of this paper, the research team considered the multi-level model as dynamic at a specific level, and dependent on the interactions between the levels in regard to DHL. The final coding scheme represented the multi-level key barriers, facilitators, and recommendations associated with digital health literacy and the broader digitalization of healthcare in cancer care as observed by CHAs [32]. The diagram generated to display the interactions with the Taplin Multi-Level Intervention model was framed within the current study to generate the model diagram based on the qualitative results (Figure 1) [31]. Demographic characteristics were collected from all interviewed participants and are presented using descriptive statistics. All analyses was conducted using NVivo Version 12 Software (QSR International, Burlington, MA, USA).

## 3. Results

**Figure 2:**
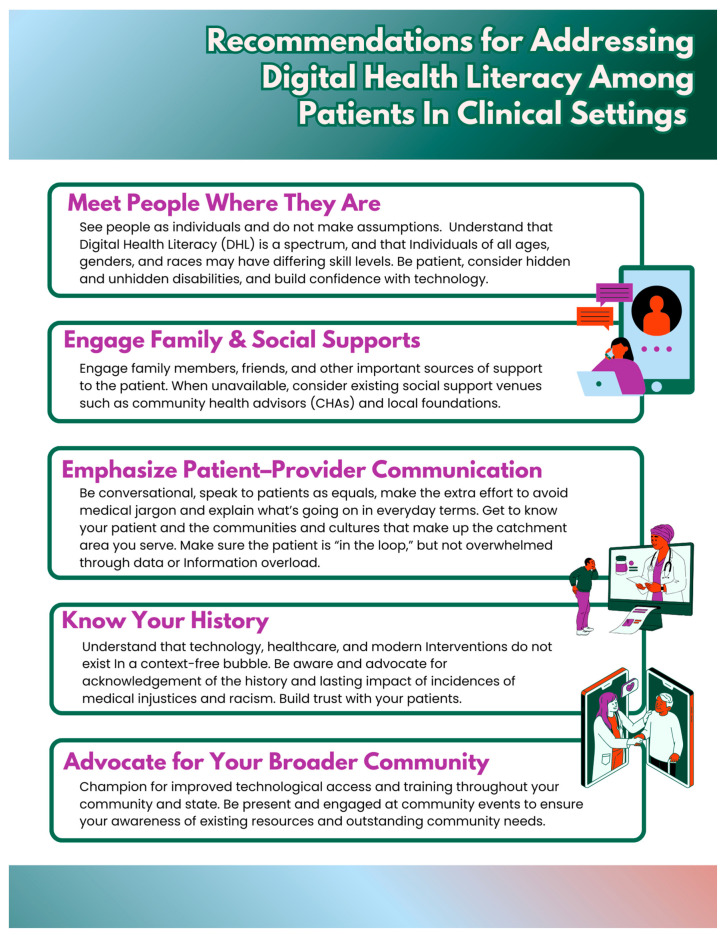
Recommendations for addressing digital health literacy among patients in clinical settings.

All thirty-three CHAs who were approached agreed to participate in the study; however, one interview did not record, and therefore, we were unable to include it in the dataset, and thus the sample was of thirty-two participants. Of the thirty-two interviewed CHAs, the median age was 64 (IQR, 59–70), 94% were female, 94% Black or African American, 28% college graduates, and 41% partnered (Table 1). Barriers and recommendations to DHL were identified by CHAs at the individual, familial and social support, healthcare provider and team, organization and practice setting, local community environment, and societal levels (Supplemental Table S1). Barriers and recommendations to address specified barriers to DHL (facilitators) that were also captured at each level of the Taplin model are presented in Figure 1. Figure 1 is based on the figure framework generated by Taplin to anchor and visualize the multi-level influences on the cancer care continuum, as they influence at the specific ecological level [31]. While Figure 1 utilizes the diagram framework from the Taplin model, the diagram presents the barriers and recommendations from CHAs at each ecological level within the visual representation. Recommendations derived from interviews with the CHAs are illustrated in an infographic format in Figure 2 for practical application of the study findings within healthcare services and cancer care interventions.

### 3.1. Individual Level

CHA-identified individual-level barriers to DHL included demographic characteristics, technological access, and personal feelings towards technology. Specific demographic characteristics mentioned by CHAs were wide ranging when describing the community that they serve in terms of age, gender, race, ethnicity, disability status, education level, socioeconomic status, and employment status. Older age was the most frequently mentioned barrier to DHL, as many believed the ability to learn and adapt to new technology decreases over time. However, CHAs argued DHL was not just an issue for older populations, but also for individuals who are historically disadvantaged communities that have been marginalized or underserved, overburdened, and have limited access to resources [33]. They discussed how in the case of healthcare visits, individuals in the Rural South would often only seek care in emergency situations and prefer to rely on “home remedies” that have been passed down from trusted sources within the community. The CHAs felt this was particularly the case for Black men, who several CHAs mentioned as being the “hardest” group to convince to seek medical attention: “The average Black man has to be mighty sick before he will go to the doctor… he [community member associated with CHA] was diagnosed with prostate cancer, he was a member of my church. And I talked to him, and he refused… The only solution to his problem was to have an operation. But he said he wasn’t going to let nobody cut on him. And he didn’t. He died because he really didn’t understand that the operation probably could have prolonged his life” (CHA 21). These experiences were emphasized in relation to “inequities because of maybe poverty or because of their ethnicity, and if one does not have access or have a low health literacy… that’s just going to make the divide between… those people who have low health literacy, as we begin to do more and more digitally, the gap is just going to get wider and wider” (CHA 4).

Furthermore, they discussed how technology access, both in terms of physical equipment and experience with technology, could impact comfort with technology in medical settings. CHAs emphasized that people in the community “do not have phones they can do it [digital health interventions] on anyway and don’t have computers” (CHA 26). Compounding this are the individual’s baseline personal feelings toward or experience with technology, including having a distrust in and being intimidated by technology. Many have a “fear of messing up” and losing important health information or getting taken advantage of by a scam. This also included being “traditional” and believing that adding technological components to healthcare was essentially “fixing something that wasn’t broken” and adding additional work for patients. CHA’s highlighted lack of experience with technology as “one more hurdle that a person has to jump over to access help” (CHA 32). Distrust in technology also underlies CHAs’ views as “technology can get it wrong a lot of times. It’s not a 100% thing” (CHA 16).

To address individual-level barriers to DHL, CHAs recommended changes in communication and interpersonal clinical interactions with patients, along with suggestions for the technological barriers. Attitude-based recommendations focused on patience, and “not meet[ing] them [patients] like I’m superior to you” (CHA 26). CHAs also emphasized that access to technology via providing phones or computers was only “the first step”. (CHA 10), and additional structural shifts were needed to ensure adequate knowledge of technology use. One CHA suggested “in-home training to senior citizens on how to use technology” (CHA 18). Another CHA suggested having “little mini session when they come into their appointments. If they see they’re struggling, trying to provide that information like confirming appointments or whatever. Or even if they just express that they don’t understand” (CHA 19). While the focus was on digital health literacy, CHAs consistently weaved in relations to basic literacy, health literacy, and general issues navigating the healthcare system. The information facilitators captured at this level and the subsequent levels is presented in Figure 1.

### 3.2. Familial and Social Support Level

At the familial and social support level, the most commonly discussed barrier to DHL was not having someone close to them that could help with technology or attend appointments with them. Many discussed having to rely on a younger member of their family to help them and noted that individuals without social support are particularly at risk. For example, CHA 20 said, “Thank goodness I got somebody that can help… Some older people don’t have a younger person to show them, or that young person won’t take the time to show them”.

To address familial and social support-level barriers, CHAs recommended active engagement with patient support networks to facilitate DHL and connecting patients to available hospital and community services and digital interventions. CHA 15 stated “they [patients] don’t understand and know how to do it [engage with digital health resources], then they’re going to be reluctant to do it… the main thing I think is just to have someone to say that, ‘Ms. Jones [de-identified community member’s name], this [digital health tool] is what we’re considering doing for our patients and we’re going to need you to do [it too]. Do you have somebody that will be able to help you do this?’ And if they don’t, then that’s where maybe your CHAs may be an avenue for support and stuff to help work with them for a short spell, not long-term, but until they feel more comfortable and learn how to do it on their own”. CHA 31 said that she “tell[s] them take somebody with you, so they can show you what is going on”, and then she also “offer[s] to go with them if they want”. CHA 16 viewed this role as “taking someone to a kiosk… [and] show them… [and answer] if they have a question about, ‘How do I use this?’ Or, ‘How do I find this out?’”. CHAs also reiterated that people “don’t realize that there are actual resources out there” (CHA 16) ranging from digital health-related information to services offered by the hospital itself or the communities.

### 3.3. Healthcare Provider and Team Level

Healthcare provider and team-level barriers were dominated by communication issues between care teams and patients, including overuse of medical jargon, improper information sharing, perceived cultural illiteracy of providers, and language barriers. Many CHAs spoke about how providers struggle to rephrase medical issues and advice into basic and straightforward language, which leads to the patient not understanding their medical issues or treatment. This occurs both in in-person conversations, and also in the medical information that is shared with patients through the portal. For example, CHA 12 said that although she knew how to access the patient portal, she did not regularly use it, because if “you do go into the digital system to read your chart, you can quickly get the wrong understanding of what is going on with your health and you might put things way out of perspective than what they really are”. In other words, it is important that patients have digital access to their health information. However, increased sharing does not always mean that there is an increase in internalization of the information, as the medium of sharing information also directs understanding of it. Another CHA relayed a story of a close friend who learned of a cancer diagnosis through results posted on their patient portal, rather than in conversation with a member of their care team. In this way, CHAs cautioned against the idea that “information is power”, because improperly shared and internalized information could not only cause increased confusion but be potentially harmful to the patient as well.

When discussing digital health tools, CHAs suggested that medical communication and cultural literacy training could be particularly useful for providers who rely too heavily on medical jargon or who are transplants to the communities in which they are practicing. Recommendations underscored the impact on DHL through language and the demeanor providers had in patient interactions. CHA 10 mentioned “if you don’t actually understand the actual language, then you’re never going to be able to really understand what’s going on with your health”. On the other hand, CHA 32 called for providers to be more understanding of the patient’s circumstances as they “listen to their patients and… advocates… [as they] don’t know what that person’s dealing with, or their past, or their family circumstances”. While these recommendations connect to the health literacy factors, CHAs mentioned “when you get a cancer diagnosis, that might be a face-to-face conversation” (CHA 32), highlighting the significance of using appropriate communication methods. This was also extended to providing written information or “spelling of that [diagnosis]” (CHA 8).

Most importantly, CHAs recommended leaning in rather than shying away from historical incidences of medical mistrust and injustices in regard to addressing DHL barriers at the provider level. This is particularly important in the context of the Deep South, where knowledge and implications of the Tuskegee Syphilis Study is widespread. CHA 32 expressed that doctors should say, “I’m aware that your people have been used and abused, that their bodies have been used and abused and mutilated… But that’s not my goal. My intention is to help you and to give you options”. Thus, acknowledging knowledge of past events created “credibility with their patients” (CHA 32) and fostered greater trust in present care, which transcends to care provided employing digital tools and interventions.

### 3.4. Organization and Practice Setting Level

Barriers identified at this level revolved around health system infrastructure and culture and included subthemes of shortage of staff, providers, and navigation programs. The primary example given here by several CHAs referred to the “kiosk” check-in system at UAB clinics and other clinics across the country. Essentially, the electronic check-in system bypasses the need for clinic staff to physically check patients into their appointments. CHAs mentioned that “when you go to the doctor now, you’re sent to a kiosk before you even get to see the doctor” (CHA 7). However, CHAs felt “the transition was too quick” (CHA 7) and that patients were not properly taught how to utilize the kiosk. The technological transition also harbored the sentiment of depersonalization at times, since there is no one “say[ing] “How are you doing?”… [patients] have to check in at a kiosk, that sometimes just drives me [CHA] crazy” (CHA 32). While they recognized that this infrastructure transition is likely necessary due to staffing and provider shortages, it also added an additional technological barrier to patients accessing care.

To address the DHL barriers at this level, CHAs underscored provider training and relationship building with local community partners on infrastructural changes associated with DHL, whilst emphasizing to patients the benefits of technology in healthcare. CHAs directly address the technological transition of facilitating care by emphasizing the role dissemination of benefits of technology has on patients. CHAs mentioned communication and cultural training integrated into healthcare infrastructure for providers, which also connects to the barriers expressed at the provider level. CHA 19 states “that personal touch… approachable conversation and they [doctors] would learn from us [CHAs] because we… know how to connect with the community on another level than what the physician does”. Additionally, when integrating technology within the healthcare system, CHAs advocated for “[having] people to help them [patients] maneuver the machines [kiosks, and healthcare associated technological tools]” (CHA 7). While this extends the roles and responsibilities of administrative healthcare staff, it also bridges the potential discrepancy in utilizing technological interventions in healthcare settings. Part of the recommendation in addressing practice setting barriers to DHL was by “bringing it down to the level where the community member can understand” (CHA 19). Additionally, CHA 23 pointed out that part of this change in healthcare infrastructure involves relaying benefits of technology in conjunction to DHL education. It equips the patient with “access to your [patient] information… [with this] you reviewed your chart, then you have more specific questions to ask” (CHA 23). This centralizes the employment of health-related technologies by patients to better empower them in healthcare interactions and in taking care of their health.

### 3.5. Local Community Environment Level

Barriers at the local community environment level centered on rurality and shared beliefs. Rurality and the associated lack of adequate cellular and internet access is a significant problem in the state of Alabama, as 55 out of 67 of Alabama’s counties are considered rural and 43.6% of Alabama’s population live in a rural area [34]. For Mississippi, 65 out of 82 counties with 54% population residing in rural areas are considered rural, along with having the lowest physician to patient ratio [35,36,37]. Many CHAs referenced a recent program began by the Alabama governor to expand high-speed internet access across the state, but noted they were unsure when and how the program would be put into effect. Shared community beliefs such as the gatekeeping of resources from historically marginalized communities, medical mistrust, and the idea that society at-large is moving virtual also impact digital health literacy. CHAs noted that “you find out about a program that will provide you transportation to and from your appointments, or a program where you can get a home health aide or a home health nurse to come into your home and you don’t have to travel, by the time we find that out, with some of us, then it might be a little bit too late” and the program no longer exists or is overloaded (CHA 24). Additionally, echoing the sentiment of other CHAs and community members, CHA 30 stated “I don’t do trial drugs…which I have heard that a lot of Black people will not do trial drugs because of those things that have happened to us in the past”. CHAs stressed that this dynamic of mistrust between the healthcare system as a whole and community environment will be strengthened when the community “see[s] the doctors… [as] people that actually care, not just dispensing medication” (CHA 23). As the CHAs were predominantly from the African American community, they centralized the medical mistrust seeped in through years of injustices towards the southern Black community.

For addressing DHL barriers at the community level, CHAs highlight the importance of human connection and a demonstrated desire to understand the community needs. For example, CHA 32 stated that healthcare providers should “come alongside them [community members] and ask them what is it they need”. Overall, to address the disconnection between communities and healthcare, CHAs not only recommended provider participation in community and assessment of community needs but linked it to DHL through community level trainings that are integrated into “different things that go on [in the community]… [such as] health events” (CHA 16). They also mentioned the role of the church in being locations for cancer screening and education or health-related technology training. CHAs also stressed the role they play as they “relate or relay messages” to the community members and provide support in “understand[ing]… medical terms or… a lot of things that the doctor tell[s]” (CHA 9). Stressing community level resources in supporting DHL barriers, CHAs encourage the facilitation of “asking for volunteers for the community or the older people” (CHA 19) when it comes to DHL education.

Acknowledging the changes brought on by the COVID-19 pandemic and society’s at-large reliance on or transition towards technology in healthcare, CHAs mentioned their hesitancies. CHAs mentioned that “it was COVID that actually exposed just how much of a barrier having technology actually was…finding out how many individuals really did not have access. Not only to the high-speed internet, but also having access to technology” (CHA 10). CHAs recommended better grasp of community level hesitancies and infrastructure needed for technological interventions in healthcare, specifically in understanding and interacting with telehealth. When discussing about telehealth and technological integration into healthcare, CHW 15 mentioned “preparing people, and sometimes we have to prepare them mentally as well as physically to do certain things”. Echoing other CHAs, this underscores the need for gradual change, education, and consistent assessment of community level needs in addressing DHL barriers at this level.

### 3.6. Illustration of Barriers and Recommendations to Address Specified Barriers to DHL

The communication tool, developed based on insights from interviews with CHAs and analysis of DHL in association to the the Taplin Multi-Level Intervention model, highlights key health systems, sociocultural, and community based considerations for DHL engagement (Figure 2). It is designed to promote informed understanding and support for patients on key aspects of DHL. 

## 4. Discussion

Community health advisors in Alabama and Mississippi highlight the importance of communication and community-based partnerships in regard to addressing patient-related barriers to DHL. Within the interplay of the multi-level factors, the CHAs iteratively emphasized serving the community as the grounding role in providing feedback and shaping DHL. Our findings compliment barriers identified in other studies [21,38,39,40], yet seven CHAs expanded on the lived experience of those barriers within the unique rural, southern context and emphasized the need for greater community engagement [41]. The Taplin Multi-Level Intervention model takes on an ecological perspective that the multiple levels influencing healthcare delivery and disparities—in this case DHL—are interdependent, with interventions and recommendations at several levels [31]. This specific consideration of DHL in patients with cancer in the Deep South fosters avenues for potential change in healthcare delivery as interactive at various Taplin levels, which goes beyond the technological instruments. As such, there is an urgent need for policies that integrate community health advisors into healthcare systems, supporting their efforts to bridge communication gaps and enhance digital health literacy in rural areas. This can lead to more effectively tailored interventions that address the unique needs of these communities.

While acknowledging that low DHL more frequently occurs within certain demographic groups, CHAs highlighted the importance of individual traits and attitudes towards technological barriers, such as a reluctance to seek help and a preference for face-to-face interactions. These personality-related factors are often overlooked within research for accessibility concerns or training-related concerns to digital or e-health literacy [21]. In studies on chronic illnesses, researchers highlight non-adherence to technology and facilitation of DHL due to perception of decreased face-to-face encounters with healthcare professionals, which was seen as further depersonalizing health management [42,43]. Thus, the impediment to digital health literacy caused by personality factors can exacerbate perceived depersonalization in doctor–patient interactions that occur in healthcare, as there is a desire for empathetic, personal, and offline attention intersecting with barriers to communication. To address this, policy reforms should ensure that digital health interventions prioritize empathetic communication and support systems that combine both digital and in-person care models, reducing patient concerns about depersonalization. For instance, with telehealth, patients have echoed concerns about errors with their physical exam, perceptions that providers are less attentive, barriers to vocalizing their questions and concerns, and difficulty in establishing a patient–provider relationship digitally [44]. This is heightened by increasing spread of health-related misinformation, along with some patients’ lack of experiences with digital access or knowledge [45,46,47].

In incorporating the CHAs’ perspectives, this study aligns with the suggestions to take a participatory approach to capture diverse and shared cultural values, beliefs, and experiences, particularly in the Black or African American community [48,49]. CHAs occupy this space between the interpersonal, community, and provider—with an ability to integrate into health systems while maintaining community level engagement and identity [50]. CHAs are key to ensuring the cultural and linguistic suitability, acceptability, usability, and effectiveness of DHL-based interventions [51]. Therefore, policies should promote the inclusion of CHAs in the design, implementation, and evaluation of digital health initiatives to ensure cultural relevance and improve patient engagement in rural communities. Inclusion of CHAs elaborates on the significant role of community engagement and support, such as churches, alongside tapping into roles existing social services play in addressing DHL. Addressing DHL in this inclusive mechanism is critical in rural Alabama, Mississippi, and the Deep South, where digitally based intervention may provide opportunities for engaging with cancer care [52,53].

The disconnect in communication between providers and patients is especially true in populations with a history of medical mistreatment and mistrust, as echoed by CHAs [54]. While digital health technologies have been shown to enhance patient–provider relationships, CHAs signify the role of DHL in this process and the community-level environment that shapes this interaction. To improve patient trust and engagement in digital health, healthcare providers and policymakers must prioritize the development of community-based digital health literacy programs, emphasizing transparency, cultural sensitivity, and patient autonomy. Aligning with the CHAs’ recommendations, Muvuka and colleagues outlined strategies for health and digital health literacy in the African American community [48]. They identified the need for tailoring information to cultural factors, community resources and integration of community pillars, literacy needs, health needs, and personal and cultural values [48]. In an adjacent qualitative study by Ali and colleagues, they noted the role community health workers play in navigating health-related literacy—by extension, DHL—as they are from or familiar with the community, trustworthy, nonjudgmental, and knowledgeable about health and digital health [55]. This integration of local infrastructure, history, and cultural environment builds on Baum and colleagues’ development of digital information and communication technologies as social determinants of health [15]. While the Taplin model reiterates interactive influences on multiple levels on DHL, Baum and colleagues emphasize the iterative and cyclic impact of digital capital on other social determinants of health that absolutely or relatively excludes individuals from benefits of technology [15]. Aligning with this significance of social environments and local connections, the CHAs propose leveraging community-based events and understanding the intersecting identities and values that shape literacy and engagement with digital resources.

Regarding limitations of this study, our results primarily represent experiences of CHAs and patients of the local Alabama and Mississippi regions. Secondly, the cross-sectional nature of the study prevents us from drawing causal inferences and restricts our conclusions to descriptions of the multi-level relationships. Thirdly, we did not measure digital health literacy in the CHAs or the patients they care for, but rather focused on their outlook on DHL and healthcare relations. This calls for future research that comparatively accounts for DHL in rural regions of Alabama, Mississippi, and across various regions of the United States, alongside the varying multi-level barriers, support factors within patients, CHAs, and provider populations. Additionally, CHAs underscore the significant role that community environment and infrastructure plays in DHL; thus, future research should mold in community-based participatory research practices, alongside inclusion of CHAs and community stakeholders as key players in the research mechanism. While we anticipate that the perspectives of CHAs will crosscut and extend beyond cancer, in our sample, their interactions with cancer patients, education, and care influenced their perspectives on DHL. Lastly, the impact of COVID-19 underscored at the local community environment level presents an avenue for future research to explore the engagements between epidemics and pandemics in light of DHL and CHAs in rural regions.

## 5. Conclusions

The interplay between the varying Taplin model levels of influence on cancer fosters feasibility and sustainability of DHL in interactions with impacts on health inequities. At the individual level, the CHAs stress the barriers tied to access to and comfort with technology. CHAs build on individual comforts at the provider and team levels through building trust via provider communication, cultural literacy, and mutual respect. For CHAs, facilitation of barriers at the organizational and practice setting levels is through leveraging training, offering resources to promote DHL, and collaborative engagement with community partnerships. At the local community environment level, CHAs highlight the influence of policy changes, active presence of healthcare system and providers to build trust and centralization of the community lens in DHL interventions. Within the Deep South, CHAs express the significant roles they and the community-situated infrastructure play in shaping DHL.

## Figures and Tables

**Table 1:** Community health advisor demographics (N = 32).

	*n* (%)
Location (Alabama or Mississippi)	
Alabama	26 (81%)
Mississippi	6 (19%)
Gender	
Female	30 (94%)
Male	2 (6%)
Age (37–82)	
Age, Median (IQR)	64 (IQR, 59–70)
30–60	10 (31%)
61 and older	22 (69%)
Race	
Black	30 (94%)
White	2 (6%)
Education	
High School	3 (9%)
Associates Degree/Technical/Vocational School/Some College	12 (38%)
College	9 (28%)
Masters/Doctoral Degree	10 (31%)
Marital Status	
Unpartnered	8 (25%)
Married/Partnered	13 (41%)
Divorced	6 (18.75%)
Widowed	5 (15.63%)

## Data Availability

A portion of the dataset is contained within the article or Supplementary Materials. Dataset available on request from the authors.

## Supplementary Materials

The following supporting information can be downloaded at: https://www.mdpi.com/article/10.3390/ijerph22010041/s1, Table S1. Perceived Barriers to Digital Health Literacy according to the Taplin Multi-Level Intervention Model.
