# Supplementary material for: Understanding Multi-Level Factors Impacting Digital Health Literacy in the Deep South of the United States
Source: Int J Environ Res Public Health. 2024 Dec 31;22(1):41. doi: 10.3390/ijerph22010041 (PMC11764728; doi:10.3390/ijerph22010041)
Supplement: Supplementary file 1 [file ijerph-22-00041-s001.zip › ijerph-3303171-supplementary.pdf]

Taplin Model Level	Factor	Barriers	Exemplary Quote	Taplin Model Level	Factor	Facilitator/ Recommendation	Quotes
**Supplemental Table 1.** Perceived Barriers to Digital Health Literacy according to the Taplin Multi-Level Intervention Model.							
Individual	Demographic	Age	"A lot of the older generation family and friends, they have very little technology skills or computer literacy skills." CHA 9	Individual	Demographic	Be Patient	"Well, be patient. Like you were patient with me when I first tried to get on [zoom to do interview]." CHA 14
		Gender	"The average Black man has to be mighty sick before he will go to the doctor." CHA 21			Meet People Where They Are	"Don't meet them like I'm superior to you. No. That's an attitude you don't never want to walk out with." CHA 26
		Race/Ethnicity	"Most White [people], they going to go and do they screening, they going to go and get stuff checked out. But with us [Black people], like I say, we don't want to go, or be afraid to go, or just slacking going to get our screens, and going to the doctor and stuff." CHA 17				
		Education Level	"And he hasn't any education. And maybe sometimes, once or twice he's been misled with information, or had			Use Large Font	"Then, especially, for example, individuals who have eye impairments and vision impairment, once upon a time, they could use that technology like it

			misleading information." CHA 31				was nothing, but because as an individual gets older, your eyes start getting a little older too, so things that you were used to doing and doing quick, it may come at a little slower pace now that you've aged and your vision has aged with you." CHA 24
		Socioeconomic Status	"We just don't have the capability or the money to afford this stuff [technology]." CHA 26			Provide Transportation	"We try to go out in the community and help people. Sometimes we might have to take a person to the doctor." CHA 26
		Disability	"I have a friend who had a stroke recently who lost the ability to swipe left and right to get to the apps. He would need assistance, if he were to have something digitally." CHA 13				
		Employment Status	"I don't even own a computer in my home because I don't work anymore. I don't use it and because I don't use it, I've lost it." CHA 30		Technology Access	Provide Technology	"I would say the first step is to make sure that access is available. And then, once access is available, we can move forward with the next steps." CHA 10

	Technology Access	Smart Phone or Computer Access	"They don't have phones they can do it on anyway and don't have computers." CHA 26			Give Non-Technology Option	"really it need to be more hand in hand like it used to be, because especially with older people. Because a lot of them don't even know a lot of stuff about technology and stuff. So to me, you need to stay with the old ways and then you could still have the new ways too." CHA 17
		Lack of experience with technology	"If you don't regularly use those things, then it's a struggle. And it is just one more hurdle that a person has to jump over to access help. " CHA 32		Feelings toward Technology	Provide Technology Assistance	"If they're not confirming their appointments or if they're not checking in, they probably need to have available some type of little training session, even on the individual basis. As they come in for their appointments, have some type of little review on their phone on how to check in and how to do this or how to save their password somewhere. But just a little mini session when they come into their appointments. If they see they're struggling,
	Feelings toward Technology	Intimidated by Technology	“I can speak to myself. I had fear of messing up when I would use technology. I was scared that, with the computer, that I would lose my information and I wouldn't be able to retrieve it if I didn't have it saved. And it's just those type fears				

			that we have.” CHA 15				trying to provide that information like confirming appointments or whatever. Or even if they just express that they don't understand." CHA 19
		Distrust in Technology	“Technology has evolved, however some things you can get it wrong with technology, and technology can get it wrong a lot of times. It's not a 100% thing.” CHA 16			Provide In-Home Technology Training	"I know one of the partners that we're working with, they provide in-home training to senior citizens on how to use technology. So, there are actually programs and modules that these individuals can complete that will show them how to use this new age technology, like sending an email or signing up for a account, and things of that nature." CHA 18
		Increased Workload for Patients	"Or having to register before you come in. And so, having to go in and put all this information in and take pictures with your phone of your driver's license, in			Provide Incentives to Using Technology	"Even though you maybe can provide some incentives, if the incentive is given at the beginning, say, when you go and get this screening or if you complete this

			order to do your doctor's appointment. " CHA 19				assessment, or this or that, you will get a $25 stipend" CHA 24
Family & Social Supports	Family & Social Supports	No or Limited Support	“Thank goodness I got somebody that can help. Just like I said, some older people don't have a younger person to show them, or that young person won't take the time to show them.” CHA 20	Family & Social Supports	Family & Social Supports	Engage Family Members and Other Avenues of Social Support	"Ensuring that they have a support. Because like you say, if they don't understand and know how to do it, then they're going to be reluctant to do it. So the main thing I think is just to have someone to say that, "… this is what we're considering doing for our patients and we're going to need you to do. Do you have somebody that will be able to help you do this?" And if they don't, then that's where maybe your CHAs may be an avenue for support and stuff to help work with them for a short spell, not long-term, but until they feel more comfortable and learn how to do it on their own." CHA 15
Provider/ Team	Doctor-Patient Communication	Overuse of Medical Jargon	“I choose not to go into MyChart to read the information	Provider/ Team	Doctor-Patient Communication	Be Conversational	"Because if you don't actually understand the actual language, then

			because I might read it wrong and get the wrong understanding of what the doctor has written down. So I will wait until I go to my provider so he can explain the information to me. So if you don't know what you are reading about, or you don't understand what you are reading about, if you do go into the digital system to read all your chart, you can quickly get the wrong understanding of what is going on with your health and you might put things way out of perspective than what they really are." CHA 12			and Avoid Medical Jargon	you're never going to be able to really understand what's going on with your health. And so, just making sure that we're using the terminology that patients can understand. That's even if we're doing educational seminars" CHA 10
						Listen More, Trust Patients, and Present Options	"If doctors would listen to their patients and listen to the advocates that come with the patients and not try to force people and make them feel bad because they have chosen not to do it the way that the doctor thinks is best. It might be best, but not best for that particular person, because we don't know what that person's dealing with, or their past, or their family circumstances." CHA 32
						Use Appropriate Communication Methods	"The doctor will say, "Well, did you see your results in the portal?" A person's like, "What

							portal? What are you talking about?" When you come into the office, or doc, they'll call you and say, "Well your results are in the portal. We just wanted to let you know." And sometimes doctors will give you your results over the phone. And I think when you get a cancer diagnosis, that might be a face-to-face conversation maybe." CHA 32
		Improper (Over or Under) Information Sharing	"When you talk to them you can't go and pull all the information out on them at one time. You got to walk them through it." CHA 14			Provide Written Information about Diagnosis and Treatment	"If I had gone in and the doctor told me, "Oh, you have phlebitis," or whatever, if I didn't understand, I would've been saying, "Okay, write that down for me," or, "Give me the spelling of that." CHA 8
						Provide Scripts or Frequently Asked Questions for Patients to Use when Speaking to Clinicians	"When you educate on them questions to ask and stuff to look for, then they're better informed. And they feel more comfortable then." CHA 31

		Cultural Illiteracy of Providers	“if you don't understand the culture, it may not come across the same if you're not familiar or not realizing you need to relate on a different way depending on the culture.” CHA 19			Emphasize Human Connection between Patient and Clinician	"Try to relate to the person you're approaching. Just don't go in quite as cold as just step up and talk. You can speak to the person first. That's what I try to do" CHA 26
						Demonstrate Knowledge of Historical Medical Injustices	"I would rather a doctor say, "I'm aware that your people have been used and abused. Their bodies have been used and abused and mutilated. But that's not my goal. My intention is to help you and to give you options." You don't have to be that graphic, but to share with their patients the truth and they're aware of it, it makes me trust you more, because you are aware of it and now I know that you're aware of it. If doctors would do that, I think it would give them more credibility with their patients. " CHA 32

Organization & Practice Setting	Health System Infrastructure and Culture	Organization Culture	“With my healthcare provider, well, not just the doctor himself, but the ones that are in between, the ones that do the scheduling and what have you, they are ... It's like, I don't know if I should say this. But they don't, well some of them, they don't really care... I can see how that could lead individuals to think that healthcare providers just don't want to talk to people anymore.” CHA 18	Organization & Practice Setting	Health System Infrastructure and Culture	Build Community Partnerships	"I think that's where we come into play as far as what we do as community health advisor. We are the people in the community where we know not all the people, but we know a good little bit of the people here. And we're able to relate or relay messages as far as they may not understand, like you said, medical terms or they may not have understood a lot of things that the doctor tell them. And we take the information and then we help them as far as scheduling appointments or they may not know the procedure or how to be screened for lung cancer. And so we give them the step by step on their level." CHA 9
						Offer Communication and Cultural	"That personal touch, because sometimes doctors are very smart, but some of them may

						Training for Clinicians	not have that charming or that approachable conversation. And they would learn from us because we, again, are called the natural helpers and we know how to connect with the community on another level than what the physician... So maybe learning how to be more approachable and a little more comforting and bringing it down to the level where the community member can understand." CHA 19
		Reliance on Technology	"One of the other thing that gets me is when you call, and this is because of modern technology. You call and you can't get a live person to answer the phone. You've got to leave a voicemail and sometimes they never call you back or whatever." CHA 21			Facilitate and Encourage Peer Education	"And even with that, sometimes even asking for volunteers for the community or the older people, they can come in and help each other. Some of them are always looking for volunteer opportunities just to sit down as they come in and help them out." CHA 19
						Emphasize Benefits of	"If the patients would understand all of your

						Technology for Patients	information is there, and now you can go and talk to your doctor and ask, "Well, what does this mean? What does that mean? Why am I being treated for this?" You do have access to your information. I guess if the patients would understand that, that it's easy to see the information for yourself. In those 15 minutes that you see your doctor, he may not have time to answer all these questions, but if you reviewed your chart, then you have more specific questions to ask." CHA 23
Local Community Environment	Rurality	No or Poor Internet Access	"I'm in a rural community and there's a whole lot of trees everywhere so it would be difficult to get a connection. And some people have problems with their cell phones in their homes, so if they can't	Local Community Environment	Rurality	Advocate and Lobby for Better Cell and Internet Access	"So, each and every year I would reach out to the cable company or whatever, to see how far they will come along to actually being able to give us the internet access that we actually needed at our house. So, by either

			use their cell phone in their homes to reach a tower, they will not be able to use the internet in their homes to reach a tower.” CHA 12				you're having someone advocating for you, that hey, say for instance that someone is in a real, rural area where there's no internet access, or anything like that, having someone being the advocate for them to say, "Well, this is why this person needs technology, because of the way that things are going, this person may need this, it can actually save their life. Say, for instance, if someone has a pacemaker, or a heart monitor." CHA 16
	Shared Knowledge and Beliefs	Perception of Gatekeeping of Resources from Historically Marginalized Communities	"We've always been considered as second class citizens, and so we were never on the front end of anything. Once you find out about a program that will provide you transportation to and from your appointments, or a program where you can get a home health		Shared Knowledge and Beliefs	Be Present at Community Events	"Her physician came and was the speaker. Stuff like that, things like that, to me really, really do serve to strengthen. I think there was more than one that actually occurred, but hers was the one I attended. I thought, "Okay, this is good. This feels helping for us to see the

			aid or a home health nurse to come into your home and you don't have to travel, by the time we find that out, with some of us, then it might be a little bit too late." CHA 24				doctors, and real people, and there's people that actually care, not just dispensing medication." CHA 23
						Ask (rather than tell) Community Needs	"But first I have to come in not trying to change them, not trying to tell them what they need, but come alongside them and asking them what is it they need. And then from there I can assess, can I help? Do I have research that can help? Which I do, but I can't walk in and give them the resources and then not walk alongside them. " CHA 32
		Medical Mistrust	"So I don't do trial drugs. I don't. Which I have heard that a lot of Black people will not do trial drugs because of those things that have happened to us in the past. " CHA 30			Provide Community Training	"Mostly the older generation that are very scared of technology, so that's something that I would like to see being done out in the community, is having different things that go on. So, say for instance that there's a health event that's going on, maybe

							a kiosk or something that's showing them how to get registered for something, or sign up for a particular program." CHA 16
						Increase Legitimacy of Telehealth	"Some things you can't spring on a person all at once. You have to prepare us in order for us to accept some things. Don't let me come here today and then you tell me the next visit I won't be seeing you anymore in-person, we're going to have to do a teleconference. We have to start preparing people, and sometimes we have to prepare them mentally as well as physically to do certain things." CHA 15
	Pandemic and Post-Pandemic Effects	Increased reliance on technology in healthcare (e.g. Telehealth)	"Because I think it was COVID that actually exposed just how much of a barrier having technology actually was. And it's from healthcare to even education and being		Pandemic and Post-Pandemic Effects		

			virtual, and finding out how many individuals really did not have access. Not only to the high speed internet, but also having access to technology at all." CHA 10				
